# Delayed surgical repair via right ventricular approach with Impella and extracorporeal life support in post-myocardial infarction ventricular septal defect: A case report

**DOI:** 10.1016/j.ijscr.2024.109417

**Published:** 2024-02-17

**Authors:** Kazuhiro Ueno, Jota Nakano, Shingo Hirao, Tatsuhiko Komiya

**Affiliations:** a Department of Cardiovascular Surgery, Oita University, 1-1 Hasamamachi Idaigaok, Yufu, Oita 879-5503, Japan; b Department of Cardiovascular Surgery, Kurashiki Central Hospital, 1-1-1 Miwa, Kurashiki, Okayama 710-0052, Japan

**Keywords:** Post-myocardial infarction ventricular septal defect, Impella, Right heart failure, Extracorporeal membrane oxygenation, Delayed surgical repair, Right ventricular approach

## Abstract

**Introduction:**

Post-myocardial infarction ventricular septal defect (PIVSD) is a life-threatening mechanical complication of acute myocardial infarction (AMI). Delayed elective surgical repair can be considered in patients who respond well to aggressive heart failure therapy. Impella has been reported as a bridge to allow the deferment of surgery for PIVSD.

**Presentation of case:**

This report describes our case of a 62-year-old male with PIVSD and cardiogenic shock. Impella was placed to ensure hemodynamic stability. Subsequently, right heart failure was suspected to be caused by insufficient circulatory support from Impella alone. Emergency surgery was considered, but it was high risk and only a few days had passed since the onset. Venoarterial extracorporeal membrane oxygenation (VA-ECMO) was implanted to treat right heart failure and delay surgical repair as long as possible. Six days after Impella implantation, the patient underwent a successful surgical repair via the right ventricle without associated adverse events.

**Discussion:**

Impella support can be insufficient for critically ill patients such as those with a larger ventricular septal defect and involvement of right ventricular function. VA-ECMO was implanted to support circulation, reduce the preload in the right ventricle, and avoid shunt inversion induced by increasing Impella flow. The patient was able to undergo a successful delayed repair with VA-ECMO at least one week after the onset of the AMI with hemodynamic stability and no associated adverse events.

**Conclusion:**

Additional VA-ECMO could help patients who fail to bridge to surgery with Impella to avoid emergency surgery, leading to successful delayed surgical repair.

## Highlights

• Impella was inserted on a patient with PIVSD as a bridge-to-surgery.
• RV failure developed even with Impella support.
• Emergency surgery was considered, but it was high risk.
• VA-ECMO was implanted to treat RV failure and delay repair surgery.
• PIVSD was successfully repaired with surgery after acute heart failure was treated.

## Abbreviations

PIVSD: Post-myocardial infarction ventricular septal defect
VA-ECMO: Venoarterial extracorporeal membrane oxygenation
LV: Left ventricular
RV: Right ventricular
AMI: Acute myocardial infarction
IABP: Intra-aortic balloon support

## 1 Introduction

Post-myocardial infarction ventricular septal defect (PIVSD) is a rare, life-threatening mechanical complication of acute myocardial infarction (AMI). PIVSD repair is associated with high mortality rates and remains challenging for cardiac surgeons. The optimal timing of surgery is also controversial. The 2017 European Society of Cardiology guidelines suggest that delayed elective surgical repair may be considered in patients who respond well to aggressive heart failure therapy [1]. The Impella has been used in AMI cases to stabilize hemodynamics. Additionally, when used as a left ventricular assist device, the Impella has been reported as a bridge to allow the deferment of surgery for PIVSD [2]. Various surgical repair techniques have been reported for the treatment of PIVSD [3,4]; the effectiveness and safety of a right ventricular (RV) approach have been recently reported [5]. Here, we report a case of successful bridging to delayed surgical repair via a RV approach using Impella and extracorporeal life support in a patient with cardiogenic shock due to PIVSD.

This case report was prepared in accordance with the SCARE guidelines [6].

## 2 Presentation of case

**Fig. 1:**
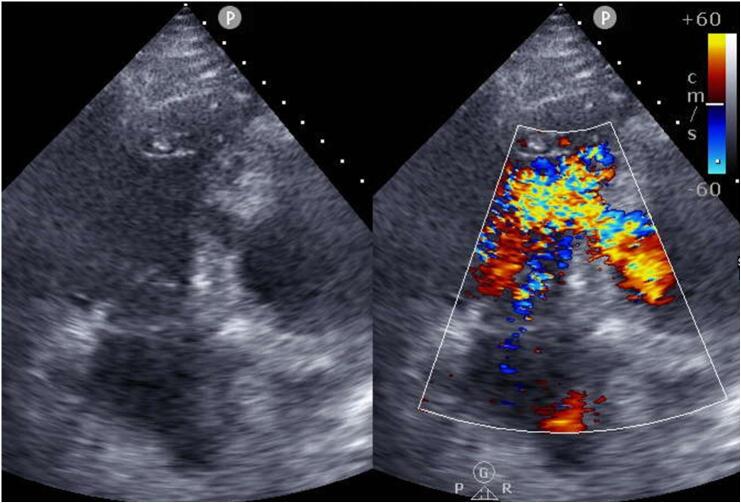
Transthoracic echocardiography. Transthoracic echocardiography shows a 10-mm ventricular septal defect (VSD) in the posterior septum.

**Fig. 2:**
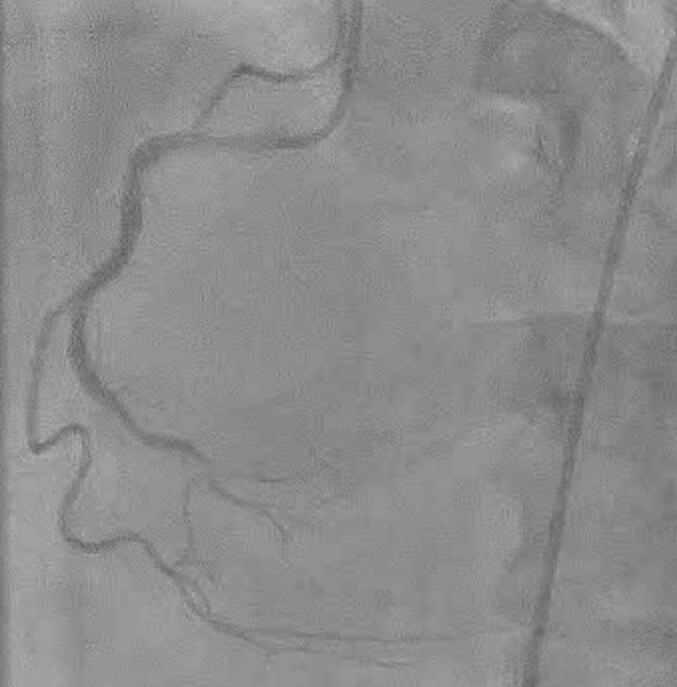
Coronary angiography. Coronary angiography showed 99 % stenosis in the distal segment of the right coronary artery.

**Fig. 3:**
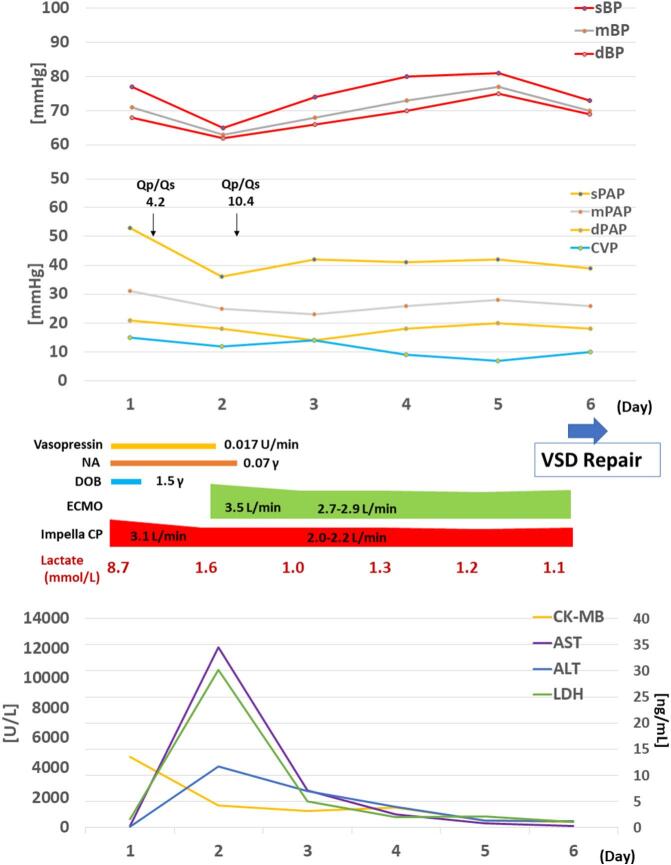
Patient clinical course. Day 1: Cardiogenic shock was complicated and Impella was initiated. Day 2: Echocardiography showed hypokinesis of the right ventricle. Central venous pressure and liver enzymes increased. Insufficient circulatory support with Impella alone resulting in right heart failure was considered. Venoarterial extracorporeal membrane oxygenation (VA-ECMO) was initiated. Days 3–5: Under Impella + VA-ECMO, circulatory status stabilized and liver enzymes decreased. Day 6: Surgical ventricular septal defect (VSD) repair via right ventricular approach was performed. Abbreviations: sBP, systolic blood pressure; mBP, mean blood pressure; dBP, diastolic blood pressure; Qp/Qs, pulmonary blood flow/systemic blood flow; sPAP, systolic pulmonary artery pressure; mPAP, mean pulmonary artery pressure; dPAP, diastolic pulmonary; CVP, central venous pressure; NA, norepinephrine; DOB, dobutamine; CK-MB, creatine kinase-myocardial band; AST, aspartate transferase; ALT, alanine aminotransferase; LDH, lactate dehydrogenase.

**Fig. 4:**
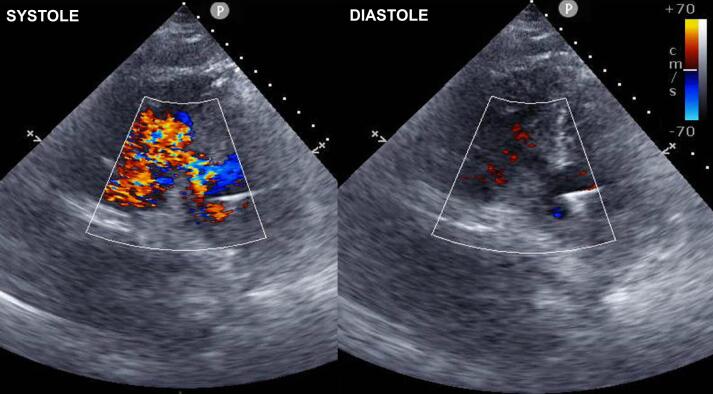
Transthoracic echocardiography before ECMO implantation. Transthoracic echocardiography shows left-to-right shunt in systole and no right-to-left shunt in diastole.

**Fig. 5:**
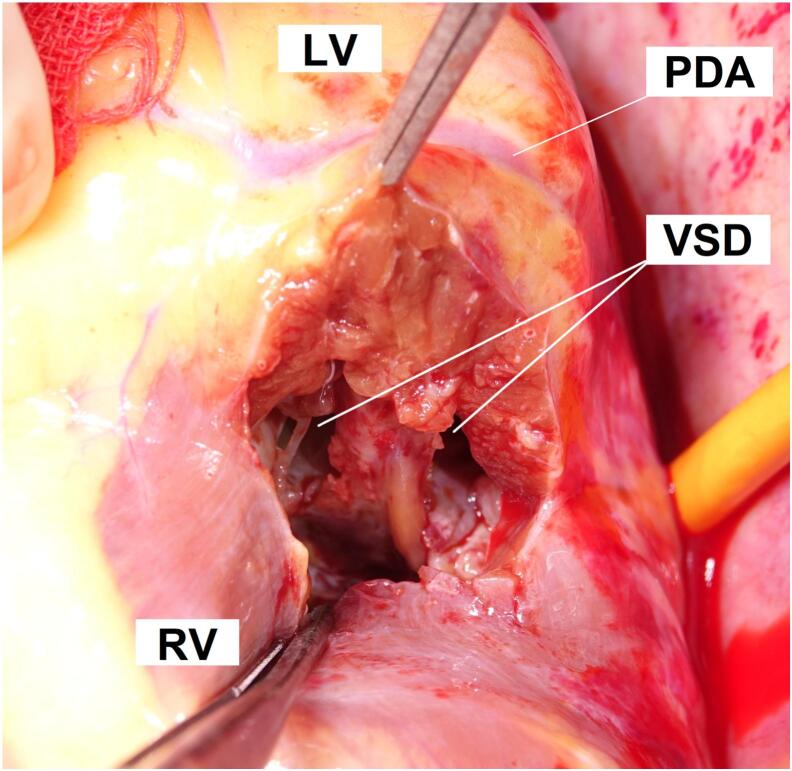
Intraoperative photographs. A right ventricular incision was made approximately 1 cm from and parallel along the posterior descending artery. A ventricular defect (1.5 cm × 2.5 cm) with a mostly necrotic tissue column was found in the apex septum. Abbreviations: LV, left ventricle; RV, right ventricle; PDA, posterior descending artery; VSD, ventricular septal defect.

A 62-year-old male presented to an outside hospital with a chief complaint of respiratory distress and was referred to our hospital with a diagnosis of PIVSD. The onset date of AMI was unknown. The systolic blood pressure was 108 mmHg, heart rate was 96 bpm of sinus rhythm, and oxygen saturation was 98 % on room air. The patient had a history of type 2 diabetes and early-onset Alzheimer's disease. The creatine kinase and creatine kinase-MB on admission were 182 U/L and 13.6 ng/mL, respectively. An electrocardiogram performed at the emergency department visit showed ST elevation in limb leads II, III, and aVF and abnormal Q waves in leads III and aVF. Echocardiography showed decreased wall motion in the posterior and inferior left ventricular (LV) wall, 40–50 % of the LV ejection fraction, relatively preserved RV wall motion with visual assessment, and a 10-mm VSD in the posterior septum (Fig. 1). The systolic blood pressure gradually decreased to 80–90 mmHg after the above examinations. Cardiogenic shock was suspected and emergency cardiac catheterization was performed. The patient was intubated in the catheterization room. Coronary angiography showed no significant stenosis in the left coronary artery and 99 % stenosis in the distal segment of the right coronary artery (Fig. 2). Percutaneous coronary intervention was not performed due to unknown date of onset. An Impella CP (Abiomed, Danvers, MA, USA) was inserted percutaneously through the right femoral artery to stabilize hemodynamics due to the relatively preserved RV function. The lactate levels decreased from 8.7 mmol/L to 4.2 mmol/L after implantation. The clinical course of the patient is shown in Fig. 3. The Impella provided a flow of 3.1 L/min at P7, and the pulmonary to systemic blood flow (Qp/Qs) ratio was 4.2 at that time. Day two after intervention, echocardiography revealed hypokinesis of the right ventricle and a dilated inferior vena cava (25 mm). There was no right-to-left shunt in diastole (Fig. 4), and PaO2 was 98 mmHg. The mean pulmonary arterial pressure was 36 mmHg and the central venous pressure increased to 15 mmHg. Laboratory data showed increased liver enzyme levels to 12,075 IU/L for aspartate aminotransferase, 4121 IU/L for alanine aminotransferase, and 10,560 IU/L for lactic acid dehydrogenase, suggesting that circulatory support with Impella alone was insufficient and resulted in right heart failure. Emergency surgery was considered, but it was high risk and only a few days had passed since the onset of the AMI. Venoarterial extracorporeal membrane oxygenation (VA-ECMO) implantation was attempted to treat the right heart failure and delay surgical repair as long as possible. The VA-ECMO was started at 3.5 L/min with 3000 ppm. Then, the Impella flow was changed from 3.1 L/min at P7 to 2.2 L/min at P4. Liver enzyme levels decreased immediately after ECMO implantation, and the hemodynamics stabilized (Fig. 3). Six days after Impella implantation, the patient underwent VSD repair. A median sternotomy was performed, and cardiopulmonary bypass was established using VA-ECMO cannulation and additional superior vena caval cannulation. An RV incision was made approximately 1 cm from and parallel to the posterior descending artery. A ventricular defect (1.5 cm × 2.5 cm) with a mostly necrotic tissue column was found in the apex septum (Fig. 5). Two bovine pericardial patches were prepared. After the necrotic column was removed, the first patch was tailored to overlap the VSD margin by approximately 1.5 cm and was sutured using 3–0 polypropylene transmurally from the LV cavity via the VSD to the RV side or to the outside of the LV. The ventricular septum appeared to be strong enough to avoid myocardial tearing by the sutures. The second patch was also fitted to the VSD on the RV side. BioGlue Surgical Adhesive (CryoLife, Kennesaw, GA, USA) was applied to the defect between the pericardial patches before the final ligation. The RV wall was closed using two Teflon felt strips for reinforcement. The ECMO was removed intraoperatively, and the Impella was left in place. The Impella flow was 2.5 L/min at P5 upon the patient transfer to the ICU. The flow was reduced to 2.3 L/min at P4 on postoperative day one and to 1.9 L/min at P2 on postoperative day three. The Impella was finally removed three days after surgery. Subsequently, thrombectomy of the bilateral femoral arteries was required for thrombi induced by long-term Impella implantation. The patient was extubated on postoperative day seven. On postoperative day eight, the patient was transferred to a general ward for rehabilitation. Although the coordination of hospital transfer was difficult due to early-onset Alzheimer's disease, the patient was transferred to a rehabilitation hospital on postoperative day 51. There were no symptoms of ischemia in the lower extremities during the hospital stay. Postoperative echocardiography revealed preserved left and RV function, no signs of residual VSD or aneurysm formation of the left ventricle, and no other significant valvular disease.

## 3 Discussion

PIVSD is a rare but life-threatening mechanical complication of AMI. The incidence is reported as approximately 0.25 % of AMI cases [7]. A VSD usually occurs in the first week after AMI. The hemodynamic consequences of VSD can lead to left and right heart failure [8,9]. PIVSD repair remains a challenge for cardiac surgeons. Perioperative mortality is extremely high at 19–54.1 % [3,10,11]. The American Heart Association 2013 guidelines recommend immediate surgery for all patients with VSD, whereas the European Society of Cardiology 2017 guidelines recommend that surgeons consider delayed repair because early surgery is a poor prognostic factor [1,12]. The survival rate of patients who undergo acute surgery has been reported as significantly poorer than that of those who undergo delayed surgery [10,13].

Intra-aortic balloon pump (IABP) is the first-line mechanical circulatory support in patients with cardiogenic shock with mechanical complications after AMI [1,12]. IABP support is, however, occasionally insufficient in more critical cases [14,15]. VA-ECMO has been used more recently for hemodynamic stabilization in patients with refractory cardiogenic shock [1,16]. Impella support has been adopted in AMI cases to improve hemodynamic stabilization by pumping blood directly from the left ventricle into the aorta, thereby decreasing myocardial wall stress [17]. Using the Impella for VSD is considered a relative contraindication; however, it has been reported as an LV assist device as a bridge to delayed surgery for PIVSD [2]. A recent study regarding mechanical circulatory support in PIVSD reported an in-hospital mortality rate for Impella of 35.3 % compared with 52 % for IABP [13]. However, Impella support can be insufficient for critically ill patients such as those with a larger VSD and involvement of RV function. Elevated Impella flow can induce a significant right-to-left shunt created by the device, leading to decreased oxygenation and hypoxia in the coronary arteries and cerebral vessels. To resolve this problem, Impella combined with ECMO has been proposed [18]. In the present case, the Impella was implanted on the day of diagnosing PIVSD. However, the patient clinical presentation revealed hypotension, and the laboratory data showed elevated liver enzymes consistent with a congestive liver, suggesting that the Impella was insufficient in supporting the circulatory system. VA-ECMO was implanted to support circulation, reduce the preload in the right ventricle, and avoid shunt inversion induced by increasing Impella flow. Although the calculated Qp/Qs ratio was increased due to the increased afterload caused by VA-ECMO, the patient was able to undergo a successful delayed repair with hemodynamic stability and no associated adverse events. The VSD repair could be performed six days after Impella implantation and at least one week after the onset of the AMI, with the infarct in the healing phase [19], as VSD usually occurs a few days after AMI, although the exact onset date was unknown due to the history of early-onset Alzheimer's disease.

The efficacy and safety of the RV approach have been recently reported [5]. In the current case VSD repair was performed via the RV approach to avoid interfering with the Impella in the left ventricle. The RV approach was feasible for VSD repair under Impella implantation.

## 4 Conclusions

Additional VA-ECMO for patients who fail to bridge to delayed surgery with Impella could be a useful strategy to avoid emergency surgery in poor conditions and lead to successful delayed surgical repair. Further studies with larger numbers of patients are needed.

## Ethical approval

Ethics clearance was not necessary as the case was judged as fitting the ethics standard criteria of our board of studies and research.

## Funding

This research received no specific grant from any funding agency in the public, commercial, or not-for-profit sectors.

## Author contribution

KU: drafting and concept; JN, SH and TK: critical revision and concept. All authors read and approved the final manuscript.

## Guarantor

Kazuhiro Ueno.

## Research registration number

Not applicable.

## Consent

Written informed consent was obtained from the patient for publication of this case report and accompanying images. A copy of the written consent is available for review by the Editor-in-Chief of this journal on request.

## Conflict of interest statement

All authors declare that there is no conflict of interest.
